# A Foot-Mounted Inertial Measurement Unit (IMU) Positioning Algorithm Based on Magnetic Constraint

**DOI:** 10.3390/s18030741

**Published:** 2018-03-01

**Authors:** Yan Wang, Xin Li, Jiaheng Zou

**Affiliations:** 1 School of Computer Science and Technology, China University of Mining and Technology, Xuzhou 221116, China; wystephen@cumt.edu.cn; 2 School of Environmental Science and Spatial Informatics, China University of Mining and Technology, Xuzhou 221116, China; zoujiaheng@cumt.edu.cn

**Keywords:** FFT, magnetic field, graph optimization, foot-mounted IMU

## Abstract

With the development of related applications, indoor positioning techniques have been more and more widely developed. Based on Wi-Fi, Bluetooth low energy (BLE) and geomagnetism, indoor positioning techniques often rely on the physical location of fingerprint information. The focus and difficulty of establishing the fingerprint database are in obtaining a relatively accurate physical location with as little given information as possible. This paper presents a foot-mounted inertial measurement unit (IMU) positioning algorithm under the loop closure constraint based on magnetic information. It can provide relatively reliable position information without maps and geomagnetic information and provides a relatively accurate coordinate for the collection of a fingerprint database. In the experiment, the features extracted by the multi-level Fourier transform method proposed in this paper are validated and the validity of loop closure matching is tested with a RANSAC-based method. Moreover, the loop closure detection results show that the cumulative error of the trajectory processed by the graph optimization algorithm is significantly suppressed, presenting a good accuracy. The average error of the trajectory under loop closure constraint is controlled below 2.15 m.

## 1. Introduction

With the development of Location Based Services (LBS), the demand for high-precision indoor positioning techniques is becoming more and more urgent. As the high accurate and reliable outdoor GPS (Global Positioning System) is not available indoors, many techniques aiming at indoor positioning have been proposed, especially the positioning techniques based on wireless sensors such as Wi-Fi, Bluetooth Low Energy (BLE) and ultra-wide bind (UWB) and the techniques based on magnetic fields and inertial measurement units (IMU). However, each technique has its own advantages and disadvantages and there is not a set of positioning techniques with significant advantages.

In recent years, indoor positioning techniques based on UWB have been rapidly developed because of its high positioning accuracy. However, although it has a decimeter-level positioning accuracy in a line-of-sight (LOS) environment [1], its accuracy in a non-line-of-sight (NLOS) environment is hard to guarantee [2,3]. Moreover, the cost of its sensor is relatively high, which is not beneficial to its application and promotion.

Positioning techniques based on magnetic fields have aroused widespread concern in recent years. Positioning is achieved by the use of the distribution difference of the magnetic field at different locations on the Earth. As there is no corresponding position space for the Earth’s magnetic field itself, the difference of the Earth’s magnetic field in space is actually caused by the difference of building structures [4]. Therefore, the fingerprint database search required for positioning with this information is relatively large and the amount of computation is also large [5].

Positioning techniques based on IMU estimate the step size and motion direction with IMU information [6]. This technique is widely applied as it does not depend on external base stations or signal features. However, this technique is only of high positioning accuracy within a short distance and its accuracy will be sharply deteriorated by the cumulative error over time [7]. Positioning through the integration of the IMU data with Wi-Fi, BLE, UWB or magnetic field information can achieve complementary advantages of the two positioning algorithms and has been widely studied in recent years [8]. By the integration of the IMU and WI-FI on the handset, the positioning error is reduced by 15% to 55% relative to the one caused by pure IMU [9] and the average positioning accuracy is about 4.5 m. The positive effect of particle filter fusion on the improvement of positioning accuracy has also been verified [10]. Besides, by combining Personal Dead-Reckoning (PDR) with BLE, a positioning result without cumulative error can be obtained only with a small number of BLE base stations [11,12]. Thanks to the high-precise and high-frequent behavioral information of the UWB sensor itself, the positioning accuracy of the pedestrian, based on the particle filter fusion UWB and the foot-mounted IMU, can reach about 1.0 m [13,14], and the UWB/INS fusion algorithm based on the EKF tight coupling makes the positioning accuracy indoor reach 0.24 m [15].

There are some studies on the integration of IMU and magnetometers. The geomagnetic and foot-mounted IMU fusion algorithm based on a Bayesian filtering algorithm achieves a long time stable positioning under the indoor scene with known geomagnetic distribution [16]. In other studies, closed-loop detection using additional generated magnetic field data is performed to ensure a steady walking trajectory up to 15 min [17]. In the field of robotic indoor navigation, the loop closure detection is carried out by the integration of odometer and magnetic field information, achieving a highly accurate indoor positioning system without dependence on external devices; besides, the absolute trajectory error in the entire process is controlled at about 0.28 m [18]. Obtaining the geomagnetic map quickly with low cost is the difficulty for the application. In the existing researches, closed-loop detection with the combination of the hand-held cell phone PDR method and the magnetometer information is used to further obtain the indoor geomagnetic distribution maps [19,20], or loop closure detection with the combination of the robot odometer and magnetometer is used to measure the distribution of indoor geomagnetic field.

These studies have achieved good results in the experiment but there are still some problems. First of all, the positioning method based on cell phone PDR is often not stable enough in practical application and only the gait change will also cause a significant increase of PDR positioning errors. Second, both in robotics-based and cellphone-based magnetic field collection algorithms, time-varying sequences are directly used to match, with a default state of uniform motion. Third, the matching algorithm heavily relies on the correct match and is too sensitive to mismatches which are hard to avoid in actual positioning.

A positioning algorithm based on foot-mounted IMU [21] and magnetometer is proposed in this paper. The position is obtained by IMU integral and the cumulative error of foot-mounted IMU positioning algorithm is suppressed by loop closure detection with magnetometer information. The main improvements of the proposed algorithm are as follows:

- Transform the magnetometer measurement from a function of time to a function of displacement, avoiding the requirements for velocity.
- Map the features to the frequency domain by FFT transform, improving the distinguishing degree of features.
- Search the most possible match with RANSAC algorithm, improving the match accuracy.
- Propose an improved loop closure error function, improving the tolerance of mismatches.

The rest of this paper is organized as follows. Feature collection of geomagnetic measured values and the algorithm for loop closure detection with the collected features are introduced in Section 2. The optimization for the trajectory obtained by foot-mounted IMU integration is outlined in Section 3 and the optimization is achieved by use of IMU, magnetometer information and pairing information provided by loop closure detection algorithms. Feature collection, loop closure detection and information fusion algorithms proposed are verified and explained in Section 4. Conclusions and prospects are given in Section 5.

## 2. Loop Closure Detection Algorithm of Geomagnetic Information

### 2.1. Feature Collection of Geomagnetic Information

In general, the space distribution of the earth’s magnetic field is fairly stable in the open environment but there will be a big difference inside the building due to the building structure. Besides, the geomagnetic signals change little with time and the magnetic field distribution in the same space position is always with high stability for a long period of time if the building structure and arrangement are not changed, which is beneficial for the loop closure detection with geomagnetic signal. However, geomagnetic information is often used to confirm direction and the geomagnetic measured values are actually direction-dependent. In order to confirm the space coordinates with magnetic field information, the three-axis raw data are usually directly compared and the original information of the magnetic field data can be well retained by this method. However, this measured value is direction-dependent, whereas the feature for loop closure detection ideally should be only relevant to its location and be independent of the motion direction. In other words, the measured geomagnetic data should present a small distance in the feature space when passing both directions of the same straight line. In the process of geomagnetic feature collection based on handheld devices and robots, this problem can be solved by adjusting magnetometer installation and using bidirectional matching. However, for the method proposed in this paper, it is difficult to fix the angle between the magnetometer direction and the motion direction due to the simultaneous binding of the IMU and magnetometer on the foot. That is, the angle between the x-axis direction of the magnetometer and the motion direction is hard to be fixed, which will cause an inevitable effect on the similarity of the measured values. Therefore, before loop closure detection, it is necessary to collect the measured geomagnetic values irrelevant to motion direction and ensure features with good descriptive characteristics are obtained.

**Figure 1:**
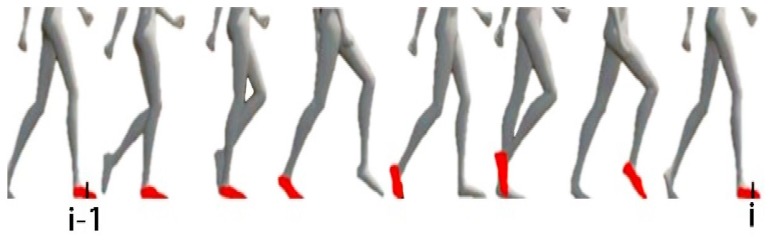
Movement status and the corresponding moment.

In the feature collection process of geomagnetic data, the measured values of geomagnetic data and IMU sensor are used simultaneously. Record the magnetometer measured values at the moment *i* as $m_{i}={\left[m_{xi},m_{yi},m_{zi}\right]}^{T}$ and the output of IMU sensor as $u_{i}={\left[a_{i}^{T},\theta_{i}^{T}\right]}^{T}$, where $a_{i}={\left[a_{xi},a_{yi},a_{zi}\right]}^{T}\in {\mathbb{R}}^{3}$ is the acceleration on three-axes and $\theta_{i}={\left[\theta_{xi},\theta_{yi},\theta_{zi}\right]}^{T}\in {\mathbb{R}}^{3}$ is the angular velocity on three-axes. It is noteworthy that, the moment *i* here corresponds to the step *i* in the positioning process. The concept of the step is shown in Figure 1. The state when the foot is completely on the ground with a velocity of 0 can be identified by the acceleration-magnitude detector [22]. The conversion of the coordinate system from step *i* to step *i−1* can be represented by $T_{i}\in SE\left(3\right)$, which can be obtained by integrating IMU observations over time. In the loop closure detection of the foot-mounted IMU geomagnetic feature, only the data when the foot is stationary on the ground can be used as a measure, as the change of geomagnetic intensity during the movement is related to the gait, which will affect the robustness of loop closure detection.

As mentioned before, the collected features of magnetic field information are required to be irrelevant to the direction and as a solution, the total magnetic field strength is used as the feature, represented by $m_{ni}$. In order to make full use of magnetometer information, the component of the magnetic field in the direction of gravitational acceleration is also used in this paper, represented by $m_{gi}$.

Corresponding relation between the magnetic field strength $m_{ni}$ and the magnetometer measured value $m_{i}$ at the moment *i* is:

(1) $$m_{ni}=\|m_{i}\|{}_{2}$$

Corresponding relation between the component on the gravity acceleration direction of magnetic field strength and magnetometer measured value at moment *i* is:

(2) $$m_{gi}=\|diag\left(0,0,1\right)R_{gi}m_{i}\|{}_{2}$$

where $m_{\mathrm{gi}}$ is the component on the gravity acceleration direction of magnetic field strength, $\mathrm{diag}\left(0,0,1\right)\in {\mathbb{R}}^{3\times 3}$ is a diagonal matrix. The rotation matrix $R_{gi}$ satisfies the following:

(3) $$R_{gi}=\arg\underset{R_{gi}}{\min}\|{\left[0,0,g\right]}^{T}-R_{gi}a_{i}\|{}_{2}$$

It is assumed here that the accelerometer coordinate system is perfectly aligned with the magnetometer one and this assumption can be easily met in practice. The static acceleration measurement is the projection of gravitational acceleration on each axis. After the rotation according to rotation matrix $R_{gi}$ in Equation (3), the z-axis direction of the coordinate system is the gravitational acceleration direction. In this way, the component of the magnetic field in the direction of gravitational acceleration is obtained.

In addition, the geomagnetic field is related to space position but in the previous methods, the sequence of magnetic field measured values is often directly compared, leading to velocity-dependent magnetic field features, as a result, magnetic field features are different when passing through the same path at different speed. In order to avoid the impact of movement velocity, firstly in this paper, the function between raw measured values and time is transformed into a function between measured values and distance. Represent the function between the position and the accumulated moving distance at the moment *i* by L(i), a series of discrete key-value pairs$\;L\left(i\right)\to m_{ni}$ and $L\left(i\right)\to m_{gi}$ can be obtained according to the time correspondence. The unit of distance is m. However, due to insufficient discrimination of geomagnetic measured values, determination of the space position depends on the variation trend of the measured values in the sequence data. In general, for the same trajectory with different movement directions, that is, passing through the same path in the positive and negative directions respectively, the variation trends of magnetic field signals are still different. Distances in the feature space are obtained by bilateral matching and the smaller one is regarded as the distance between the two moments. This distance function is defined as follows:

(4) BDD(i,j,d)=min(DD(i,j,d),IDD(i,j,d))

where min( ) is the smaller value. DD(i,j,d) and IDD(i,j,d) can be respectively defined as follows:

(5a) $$\begin{array}{c} DD\left(i,j,d\right)=w_{g}\left(inter_{g}\left(L\left(i\right)-d:L\left(i\right)+d\right)-inter_{g}\left(L\left(i\right)-d:L\left(i\right)+d\right)\right)+\\ w_{n}\left(inter_{n}\left(L\left(i\right)-d:L\left(i\right)+d\right)-inter_{n}\left(L\left(i\right)-d:L\left(i\right)+d\right)\right) \end{array}$$

(5b) $$\begin{array}{c} IDD\left(i,j,d\right)=w_{g}\left(inter_{g}\left(L\left(i\right)+d:L\left(i\right)-d\right)+inter_{g}\left(L\left(i\right)-d:L\left(i\right)+d\right)\right)+\\ w_{n}\left(inter_{n}\left(L\left(i\right)+d:L\left(i\right)-d\right)+inter_{n}\left(L\left(i\right)-d:L\left(i\right)+d\right)\right) \end{array}$$

where L(i)−d:L(i)+d is the sequence for a fixed interval, L(i)+d:L(i)−d in Equation (5b) is a descending sequence in reverse order.

In order to get a continuous function for the Fast Fourier Transform (FFT) next, the original value is required to be quadratic-spline interpolated into a continuous function. The function corresponding to$\;L\left(i\right)\to m_{gi}$ is:

(6) $$m_{gi}=inter_{g}\left(L\left(i\right)\right)$$

The function corresponding to $L\left(i\right)\to m_{ni}$ is denoted as:

(7) $$m_{ni}=inter_{n}\left(L\left(i\right)\right)$$

Then an observation value after group transform is obtained and it is independent of direction and movement velocity.

The collected frequency-domain features contain frequency and phase information, which means that a distance in feature space independent of positive or negative direction can be obtained by only once comparison. Select a path whose midpoint time is moment *i* and with a distance of 2*d*, then transform the measured values on this path and the features obtained are:

(8) $$fft_{gi}\left(d\right)=FFT\left(inter_{g}\left(L\left(i\right)-d:L\left(i\right)+d\right)\right)$$

and

(9) $$fft_{ni}\left(d\right)=FFT\left(inter_{n}\left(L\left(i\right)-d:L\left(i\right)+d\right)\right)$$

where L(i)−d:L(i)+d is a sequence for a fixed interval as mentioned above. FFT() represents the fast Fourier transform function and the result is the mapping of this sequence in frequency domain.

Furthermore, the distance in the feature space of the measured value corresponding to the specific moment can be simply expressed as:

(10) $$FD\left(i,j,d\right)=w_{g}\|fft_{gi}\left(d\right)-fft_{gj}{\left(d\right)\|}_{2}+w_{n}\|fft_{ni}\left(d\right)-fft_{nj}{\left(d\right)\|}_{2}$$

where ${\left\|˙\right\|}_{2}$ represents the modulo function and $w_{g},w_{n}$ are corresponding weight of $m_{g},m_{n}$, respectively. It is noteworthy that the weights here are with the same meaning of the ones in *BDD (i, j, d*) and are with the equal value in the experiment.

However, single FFT causes a large amount of information loss. Because the FFT represent the original signal through the sum of a series of sinusoid signal with different frequency, strength and phase offset but the information of the series of sinusoid signals’ location has been ignored in this process. In order to keep the details as much as possible, multi-level sampling FFT is proposed in this paper to keep the original data information. In this way, the distance between the two measured values in the feature space can be directly expressed as the following:

(11) $$MFD\left(i,j,D\right)=\underset{d_{k}\in D}{\sum }FD\left(i,j,d_{k}\right)\;$$

where D represents a set, which contains each sampling distance of the multi-level sampling and $d_{k}\in D$ is the *k*th value in this set. In practice, data feature can be well characterized when only 2 to 3 elements are contained in D, that is, data feature can be well characterized by the features obtained through FFT at 2 to 3 levels. By the transformation above, the distance in the feature space of the corresponding feature is obtained. Comparison of the performance between the two different distance metrics BDD and MFD will be provided in the experimental part. The algorithm of loop closure detection based on feature distance will be discussed in the next section.

### 2.2. Geomagnetic Matching Algorithm Based on RANSAC Algorithm

The original information of the feature is kept as far as possible through the multi-level Fourier transform. However, due to the intrinsic weakness of the geomagnetic distribution feature, distances in the feature space of features corresponding to different positions are still close. Besides, due to the accuracy and sampling rate of the sensor, features corresponding to the same position may also vary greatly. To solve this problem, comparison of a continuous displacement is used in this paper in order to improve the matching accuracy. Since only the geomagnetic data when foot velocity is 0 is used, the amount of data is relatively small and the distance in the feature space between all the corresponding time instant features can be directly calculated.

The feature distances corresponding to all time instant features are stored in a two-dimensional matrix. The row number and column number of the matrix represent the two time instant number before and after and the element value of the matrix represents the corresponding distances. To facilitate the following discussion, the feature distance matrix is denoted as M. The element values of the matrix are symmetric along the major diagonal axis because of the symmetry of M, so only half of the elements are required to be considered during calculation.

**Figure 2:**
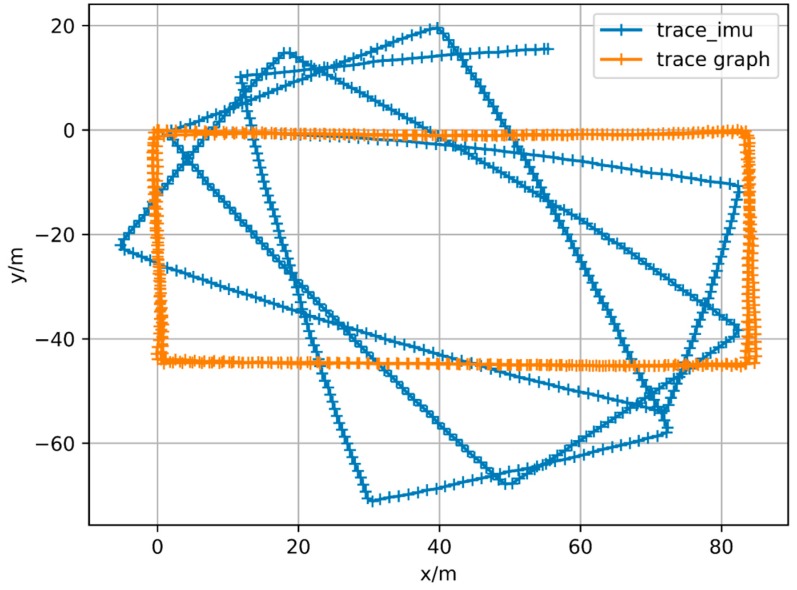
Reference Trajectory.

**Figure 3:**
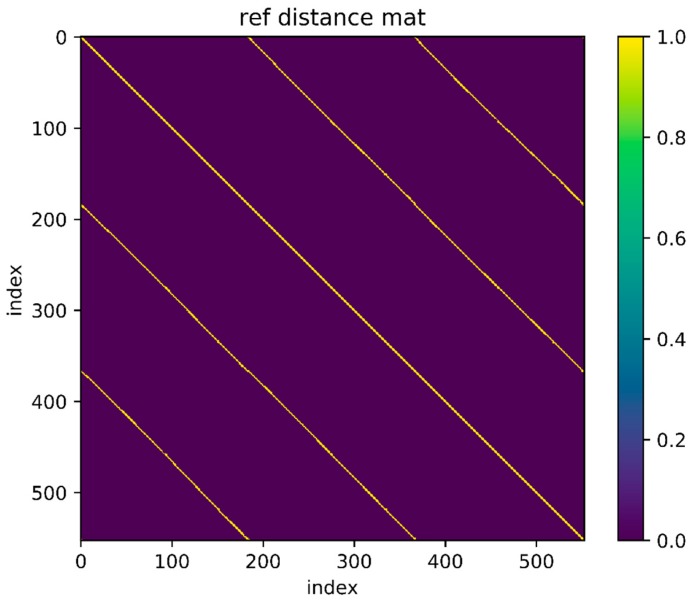
Reference distance in position space.

**Figure 4:**
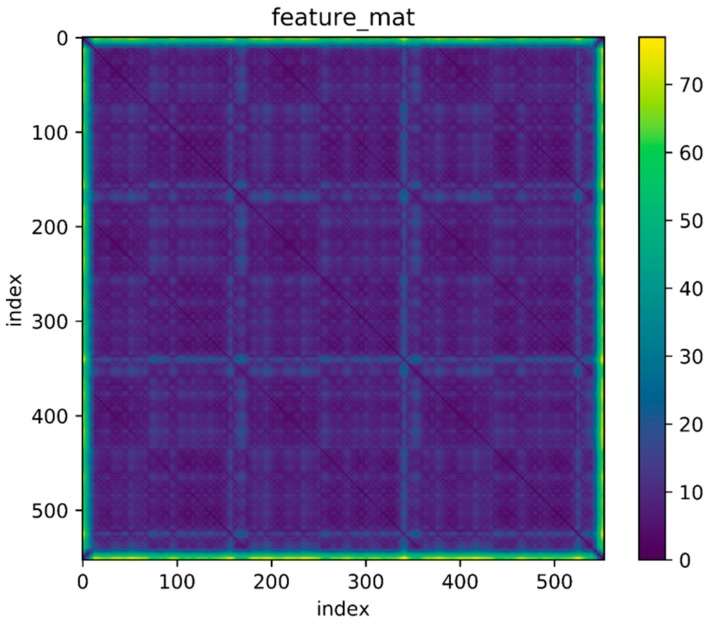
Distance of feature.

Then use an experimental result as an example. The reference trajectory of the experimental data is shown in Figure 2, three times along a matrix, that is, every point on the trajectory has been passed three times. The orange one is the reference trajectory and the blue one is the direct integral result of the foot-mounted IMU under zero-velocity constraint. The trajectory used in the experimental and the reference trajectory are obtained by setting the loop closure constraint with the same starting and ending points. Figure 3 shows a two-dimensional matrix, the horizontal and vertical axis represent the time instant. This matrix is obtained by binarization of the distance matrix, with a binarization threshold of 2 m. The distance matrix is constructed by calculating the distance between each moment based on a reference trajectory. This means that the distance between the physical locations of the two time instants represented by the yellow point’s horizontal and vertical coordinates is less than 2 m. Each point on the diagonals is in yellow as the physical distances between the moment and its several adjacent ones are close. Figure 4 shows the feature distance matrix M and the horizontal and vertical axes represent the time instant. The color of each point in the figure represents the corresponding feature distance between two time instants and the specific relationship between color and distance is shown by the color bar on the right. From the points with short physical distance presented in yellow in Figure 3, it can be found that the feature distances in the feature space corresponding to these points are also relatively close to each other, as shown in Figure 4.

**Figure 5:**
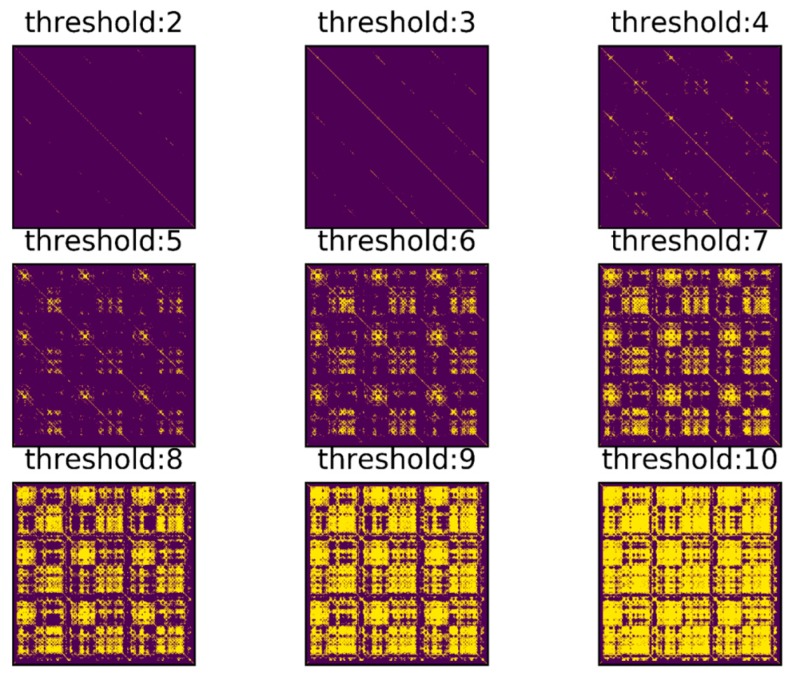
Images of selected points under different threshold.

**Figure 6:**
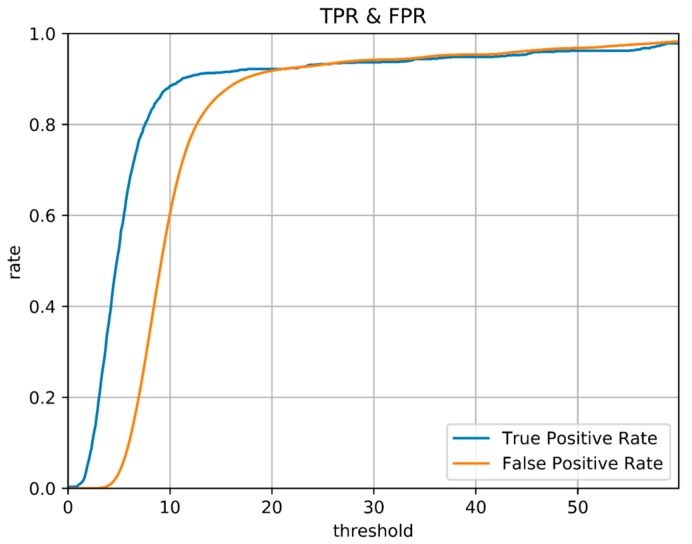
True positive Rate and False Positive Rate of different threshold.

In order to find the match relationship before and after, a straightforward method is to set a threshold and select the point whose distance is less than the threshold as the correct match. However, even for a trajectory as simple as this one, it is difficult to confirm the matching correctness by setting threshold only. Figure 5 shows a series of binary images obtained under different thresholds. Different binary matrices are obtained by setting different feature distances as the thresholds. The yellow points in the figure represent that the feature distances are less than threshold. It can be found that none of the images contains zero or a small number of false matches when a large number of correct matching points are retained. To better illustrate the effect of thresholds, the relationship between threshold and True Positive Rate (TPR) and the relationship between threshold and False Positive Rate (FPR) are shown in Figure 6. The TPR is calculated as the ratio between the number of positive samples rightly categorized as positive and the total number of actual positive samples. The FPR is calculated as the ratio between the number of negative samples wrongly categorized as positive and the total number of actual negative samples. Obviously, a proper threshold cannot be determined to achieve a very high TPR or a very low FPR. In addition, even for the trajectory with relatively obvious geomagnetic features here, the proper threshold is difficult to be determined, let alone for a more complex situation. A better approach is to use continuous matching to improve the accuracy of loop closure detection. In other words, there is a one-to-one correspondence between time instants when the same path is passed through by the trajectories before and after and this one-to-one correspondence can be used to improve the matching accuracy.

**Figure 7:**
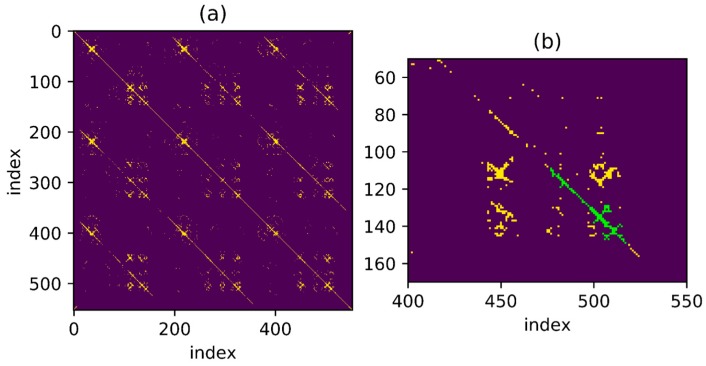
Pairs of distance in feature space smaller than threshold. (**a**) Image of the whole process; (**b**) A typically connected region.

To detect as many loopbacks as possible, set a threshold which is not too harsh at first, as shown in Figure 7a. The one-to-one correspondence mentioned above is shown in the figure as a series of points continuously distributed over a thin line and the distance between points is less than threshold. It is noteworthy that the corresponding line looks straight as the trajectory used in the example presents a relatively uniform velocity throughout the movement but for non-uniform trajectory, the result may be a curve. The goal is to find such points distributed over a continuous line in the binary result under a given threshold. Besides, in Figure 7b, for the point set marked in green, some points around the thin line are also connected to the line and an algorithm is necessary for searching the correct line under the interference of these noise points.

Find the connected region in the matrix under threshold setting and then denote the K points in the connected region as $\left(Px_{k},Py_{k}\right)$, where the value of $P_{x_{k}}$ and $P_{y_{k}}$ is sequence number. A typical connected region is shown in Figure 7b. A connected area can be defined as: if there is a line between any two points in an area and all points on the line belong to the area, then the area is a connected area. A continuous line is required to be found when a connected area is available. Intuitively speaking, the purpose is to find a linear model:

(12) L(Py)=a L(Px)+b 

where L() shows the relationship between sequence number and movement distance, as mentioned above. a,b are parameters of the linear model. If the noise is Gaussian, the optimization problem can be directly solved and proper model parameters can be obtained as follows:

(13) $$\left(a,b\right)=\arg\underset{a,b}{\min}\overset{K}{\underset{k}{\sum }}\|L{(Py_{k})-aL\left(Px_{k}\right)-b\|}_{2}$$

However, in general the typical form of a point set is shown as the green part in Figure 7b and obviously the noise does not meet the Gaussian model. A linear regression method based on the Random Sample Consensus (RANSAC) [23] can be used to find the continuous matching points. RANSAC possesses the ability to eliminate the influence of abnormal points on the model parameters and can find a proper model under the interference of a large number of noise points, which can solve the feature matching problem in this paper. With RANSAC method, the model is updated by iteration and the estimated model is obtained when it is determined that getting outlier is not affected by the outlier. Accordingly, the optimization problem can be written as:

(14) $$\left(a,b\right)=\arg\underset{a,b}{\min}\overset{K}{\underset{k}{\sum }}\omega_{k}\|L\left(Py_{k}\right)-aL\left(Px_{k}\right)-{b\|}_{2}$$

where $\omega_{k}$ indicates whether the corresponding data is outlier. Update the formula to:

(15) $$\omega_{k}=\{\begin{array}{c} 1.0\|L\left(Py_{k}\right)-aL\left(Px_{k}\right)-{b\|}_{2}<threshold_{outlier}\\ 0.0\|L\left(Py_{k}\right)-aL\left(Px_{k}\right)-{b\|}_{2}\geq threshold_{outlier} \end{array}$$

where $threshold_{outlier}$ can be specified manually or determined based on the variance of the data.

By iteration according to Equations (14) and (15), a sequence which better matches the one-to-one correspondence is obtained. In order to further reduce the false match rate, some empirical rules are necessary to improve the match, as follows:

(a) From Equation (12), the model parameter a represents the ratio between the path displacements before and after. The absolute value of a should float up and down at 1.0, as the correspondence before and after are the same physical location and the float range can be defined as a threshold, represented by $threshold_{a}$.
(b) To avoid the mismatched interference as far as possible, some matches which are too short should be given up. Denote he minimum length as$\;threshold_{len}$.
(c) The proportion of outliers in the total point set should be less than a certain threshold, that is, the matching result with the number of outliers exceeding the threshold should be discarded. This threshold is denoted as $threshold_{rate}$. The reason is that if the number of outliers in a match is too large, the feature of this trajectory will not be obvious enough, that is, the variation of magnetic field signal with the position change is not obvious. The reliability of such matching is not high enough, reflected in the feature distance map, that is, there are many mismatches around the correct match.

**Figure 8:**
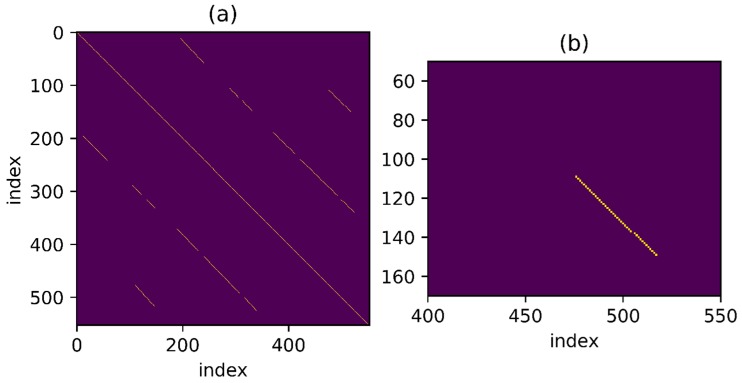
Pairs selected through RANSAC method. (**a**) Pairs selected in the whole process; (**b**) The model-match processed results of the typically connected region in Figure 7.

Figure 8a shows the matching result processed by the above algorithm. As the movement velocity of the three trajectories is relatively stable, the matching represented by the yellow line in figure is a straight line with absolute value of slope close to 1 and the one-to-one correspondence is easy to be identified. Figure 8b shows the model-match processing results of the points in the connected areas marked in green in Figure 7b. Obviously, after threshold processing, the noise points are well excluded by the algorithm and the real one-to-one correspondence is achieved.

In this chapter, a matching result with good robustness has been obtained. Next, the detected matching relationship will be used to confine the trajectory and eliminate the cumulative error of the trajectory.

## 3. Description of State Estimation Algorithm

### 3.1. Basic Formulation

The state estimation method is widely used in many fields, and the traditional one is mainly based on the Kalman Filter (KF) [24] and the Particle Filter (PF) [25] of the Bayesian filter. With the increasing demand for accuracy and the increasing computational ability of computing equipment, batch and nonlinear optimization-based techniques have become more and more important in the field of state estimation [26,27,28]. Especially for the application of eliminating cumulative error based on loop closure detection, the method based on nonlinear optimization has good universality.

Graph optimization is a typical state estimation algorithm based on nonlinear optimization. Next regarding the graph optimization, a brief description is presented, the principle is introduced and the key process is explained. Further details can be referred to in papers by Rainer Kummerle et al. [27].

In graph optimization, define the system state sequence:

(16) $$X={\left[X_{1}^{T},\ldots ,X_{n}^{T}\right]}^{T}$$

where $X_{i}\in se\left(3\right)$ is a six-dimensional vector, which represents the system state at the moment *i*. In this paper, it represents the pose of the foot-mounted IMU at the moment *i*. For the convenience of the following description, define:

(17) $$X_{i}={\left[\eta_{i}^{T},\varphi_{i}^{T}\right]}^{T}$$

where $\eta_{i}=\left[x_{i},y_{i},z_{i}\right]\in {\mathbb{R}}^{3}$ represents the three-axes translation in the world coordinate system and $\varphi_{i}^{T}\in {\mathbb{R}}^{3}$ represents the rotation in the world coordinate system. In the description process, another representation for the system state is used, denoted as $F_{i}\in SE\left(3\right)$; it is a four-dimensional matrix and its corresponding system state is $X_{i}$. The two matrices can easily be interchanged with each other and both indicate a same system state. Particularly, another repstation for rotation is also used, that is, the rotation matrix $R_{i}=\exp\left(\varphi_{i}^{\Lambda }\right)\in SO\left(3\right)$.

(18) $$\mathrm{Cos}t\left(X\right)=\sum e_{ij}^{T}\Sigma_{\mathrm{ij}}^{-1}e_{ij}$$

where $e_{ij}$ is the error function on the constraint between state $X_{i}$ and $X_{j}$. It can reflect the conformity degree of the state to the constraint and the smaller the value, the higher the conformity degree is. $\Sigma_{ij}$ is the covariance matrix, reflecting the confidence degree of this constraint. Therefore, the optimal estimated value $X^{*}$ of the state sequence can be defined as:

(19) $$X^{*}=\arg\underset{x}{\min}\sum e_{ij}^{T}\Sigma_{\mathrm{ij}}^{-1}e_{ij}$$

From the perspective of probability, this process is to solve the maximum a posterior estimation of the system state. Since the cost function used for constraints is often not a linear one, the solving process is actually an iterative linearization and a process of finding local minima. Define the state value of current system as $X_{op}=X^{*}$ and the state increment as $\Delta X^{*}$. The goal is to find the locally optimal system state increment $\Delta X^{*}$ near this point, satisfying the following:

(20) $$\Delta X^{*}=\arg\underset{\Delta X}{\min}\sum e_{ij}^{}{\left(X_{op}+\Delta X\right)}^{T}\Sigma_{ij}^{T}e_{ij}\left(X_{op}+\Delta X\right)$$

By the linearization around $X_{op}$, approximating the original error function $e_{ij}\left(X\right)$ with the first-order Taylor expansion:

(21) $$e_{ij}\left(X_{op}+\Delta X\right)\approx e_{ij}\left(X_{op}\right)+J_{ij}\Delta X\;$$

where $J_{ij}=\frac{\partial e_{ij}}{\partial X}{│}_{X_{op}}$, which is the Jacobian matrix of the error function $e_{ij}$ at $X_{op}$. Taking (6) into (5), the problem to be solved is transformed into:

(22) $$\Delta X^{*}=\arg\underset{\Delta X}{\min}\sum {\left(e_{ij}\left(X_{op}\right)+J_{ij}\Delta X\right)}^{T}\Sigma_{ij}^{-1}\left(e_{ij}\left(X_{op}\right)+J_{ij}\Delta X\right)$$

Since the partial derivative at extremum is equal to 0, linear equations on ΔX are obtained:

(23) HΔX=−b

where $H=\sum H_{ij}=\sum J_{ij}^{T}\Sigma_{ij}^{-1}J_{ij}$ and $b=\sum b_{ij}=\sum^{\text{​}}e_{ij}^{T}\Sigma_{ij}^{-1}J_{ij}$. Solve this linear equation to get $\Delta X^{*}$ and then update the estimated value $X^{*}$ of the system state according to Equation (24):

(24) $$X^{*}=X_{op}+\Delta X^{*}$$

**Figure 9:**
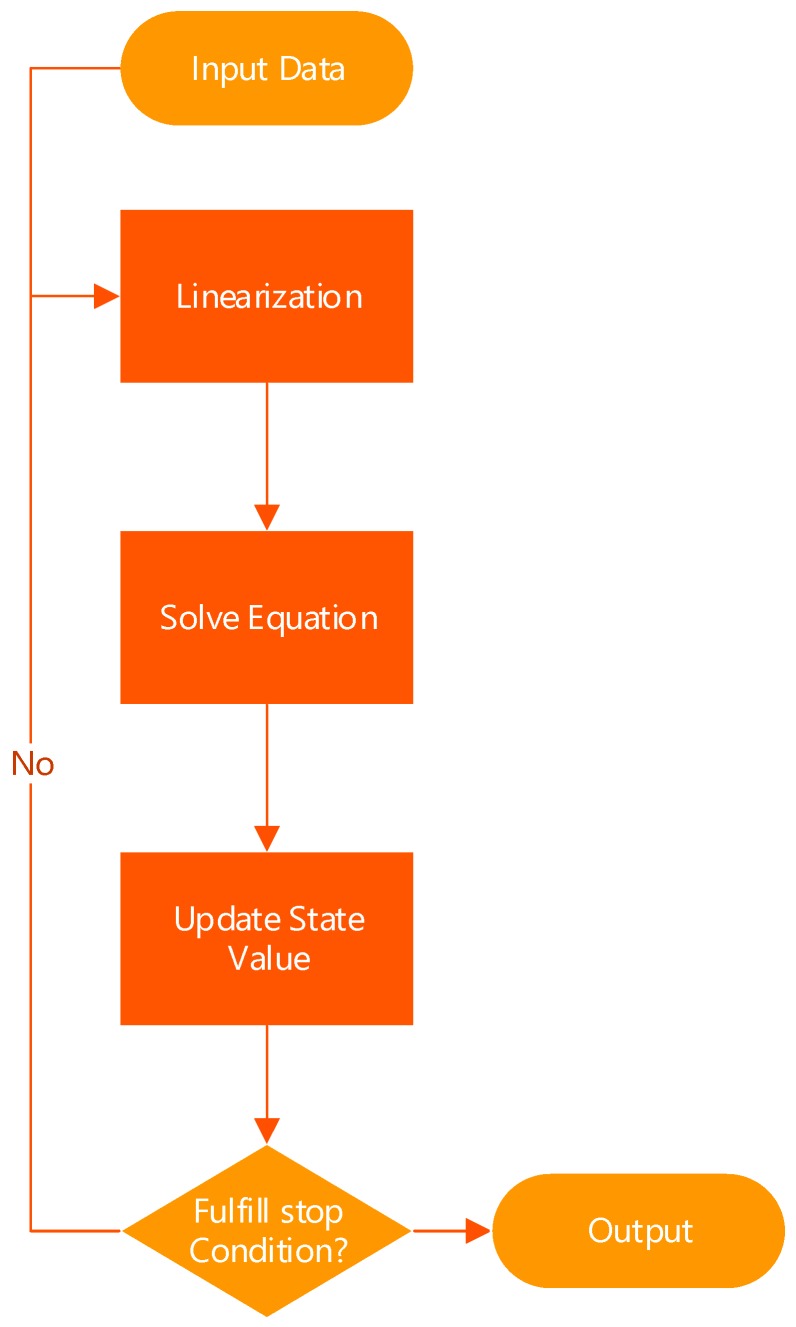
The Flow chart of Optimization.

The optimal estimation of the system state can be obtained through continuous iteration. It is noteworthy that, all computations in the previous text are required to be performed in Lie algebras [29] since the system state is represented in SE(3). The entire calculation process is shown in Figure 9. The Linearization step corresponds to the previous Equation (21), linearizing the overall error function around the operating point by a Jacobian matrix. The Solve Equation step corresponds to Equation (23), solving the linear equation in the linear algebra library and getting the update value of the state. The amount of computation is not very large as the matrix H here is actually a sparse one. The Update step corresponds to Equation (24), updating the system state $X^{*}$ with the results got from the previous step. The conditions for fulfill stop mainly include two aspects: first, whether the number of iterations has exceeded the given maximum number; second, whether the update value has been less than the threshold. Once either of them is satisfied, stop the iteration and output the current state value as the result.

In this algorithm, different error functions are required for different constraints and the error functions are directly related to the quality of the final result. Constraints based on IMU integral are obtained by integrating the IMU measurement values and the limitation that the starting and ending velocity of each step are 0 is also taken into account. If there is only one such constraint in the error function, the trajectory obtained will be consistent with the one from the EKF algorithm based on foot-mounted IMU. Constraints on magnetic and gravitational direction are common to suppress the direction cumulative error. By use of the output of the loop closure detection algorithm mentioned earlier, the loop-back constraint based on geomagnetic signals can eliminate the cumulative error caused by IMU integral, thereby improving the overall positioning accuracy. Regarding these constraints, the corresponding error function for each constraint and the Jacobian matrix for each error function are introduced in the rest of this chapter.

### 3.2. IMU Attitude Constraint

The relative pose of two consecutive time points can be obtained by time integral of IMU. Definitions of IMU measurements have been given in the text before. The system state can be obtained by time integral of $u_{t}$. $u_{i}$ represents the measurements of acceleration and angular velocity of IMU at the moment *i*, as defined above. In the traditional positioning algorithm based on foot-mounted IMU, the system velocity is constrained by the zero-velocity state of the foot-mounted IMU (that is, the state when the foot is stationary on the ground), thereby suppressing the cumulative error and improving the positioning accuracy. In this paper, the starting time point of zero-velocity state acts as the key time point of the system state and the system state $X_{i}$ at the moment *i* is actually the system state at the step *i* calculated from the initial positioning. In this way, the direct dependence of the IMU integration process on the initial state $X_{0}$ is eliminated and the system state is only related to the previous one $X_{i-1}$ and the IMU sensor observations between the two states. As a result, the method called pre-integration in visual positioning [30] is avoided and the amount of calculation is greatly reduced; moreover, the parallel computing is much easier to be realized.

The relationship of the system states between the moment *i* and the moment *i−1* can be expressed as:

(25) $$F_{i}=T_{i}F_{i-1}$$

where $T_{i}\in SE\left(3\right)$, as mentioned above, represents the coordinate system transformation between the two time instant and can be obtained by integrating the output of IMU sensor between the two time instants.

The error function can be defined as:

(26) $$e_{ins}\left(F_{i-1},F_{i}\right)=\ln{\left(T_{i}F_{i-1}F_{i}^{-1}\right)}^{\vee }\;$$

where $\ln{\left(\;\right)}^{\vee }$ represents the mapping from SE(3) to se(3). Obviously, $e_{ins}\left(F_{i-1},F_{i}\right)\in {\mathbb{R}}^{6}$. The corresponding covariance matrix is defined as:

(27) $$\Sigma_{ins}=\left[\begin{array}{cccccc} \sigma_{x} & 0 & 0 & 0 & 0 & 0\\ 0 & \sigma_{y} & 0 & 0 & 0 & 0\\ 0 & 0 & \sigma_{z} & 0 & 0 & 0\\ 0 & 0 & 0 & \sigma_{roll} & 0 & 0\\ 0 & 0 & 0 & 0 & \sigma_{pitch} & 0\\ 0 & 0 & 0 & 0 & 0 & \sigma_{yaw} \end{array}\right]$$

where $\sigma_{x},\sigma_{y},\sigma_{z}$ is the variance of measured translation value and $\sigma_{roll},\sigma_{pitch},\sigma_{yaw}$ is the variance of measured rotation value.

The corresponding right Jacobians of SE(3) is expressed as:

(28) $$J_{ins}\left(F_{i-1}\right)=\frac{\partial e_{ins}\left(F_{i-1}\right)}{\partial X_{i-1}}=-Ad\left(F_{k}F_{k-1}^{-1}\right)$$

where Ad( ) represents the transformation from rotation matrix Ad( ) to matrix ${\mathbb{R}}^{6\times 6}$.

### 3.3. Constraints in the Magnetic and Gravity Direction

In general, the displacement error of IMU integration algorithm based on ZUPT is relatively small but the direction error will be accumulated, resulting in a relatively large positioning error. The introduction of two constraints respectively on the gravitational acceleration direction and the earth’s magnetic field direction can suppress the accumulation of directional error to a certain extent. Consider that the internal magnetic field in the building is affected by the building structure and the reliability of the available absolute direction is limited, the relative constraint is mainly used in this paper. Besides, in order to reduce the amount of calculation and improve the reliability of constraint, the error function with magnetic force and gravity constraint is used to constrain the direction error only when the magnetometer observations at moment *i* and moment *j* are close. An assumption is made here that the interference of the system noise (such as buildings, electromagnetic equipment) on the magnetic field is similar at the two time instants when the magnetometer observations are close. Notably, the space locations of these time instants are not necessarily the same but the orientation of the sensor in the world coordinate system should be close.

Define the magnetometer observation at moment *i* as $m_{i}={\left[m_{x,i},m_{y,i},m_{z,i}\right]}^{T}\in {\mathbb{R}}^{3}$. As mentioned above, the magnetic field distribution in the world coordinate system should be similar when the magnetometer signals at both moments are similar. In other words, the magnetometer measurements at the moment *i* and the moment *j* should be equal after conversion to the world coordinate system. Based on this, the error function is defined as follows:

(29) $$e_{mag}\left(R_{i},R_{j}\right)=R_{i}\frac{m_{i}}{\|m_{i}{\|}_{2}}-R_{j}\frac{m_{j}}{\|m_{j}\|{}_{2}}$$

where $\|\;{\|}_{2}$ represents the *L2* norm of the vector. $R_{i}$ and $R_{j}$ are the rotation matrices corresponding to $T_{i}$ and $T_{j}$, respectively.

Similarly, the error function based on the gravity direction can be defined as:

(30) $$e_{gravity}\left(R_{i},R_{j}\right)=R_{i}a_{i}-R_{j}a_{j}$$

where $a_{i}$ and $a_{j}$ are the acceleration measurements at moment *i* and moment *j*, respectively.

In general, the Jacobian matrix can be obtained with a perturbation model. The derivative of the constraint on magnetic field direction is:

(31a) $$J_{mag}\left(R_{i}\right)=\frac{\partial e_{mag}\left(R_{i},R_{j}\right)}{\partial \varphi_{i}}=-{\left(R_{i}\frac{m_{i}}{\|m_{i}\|{}_{2}}\right)}^{\Lambda }$$

(31b) $$J_{mag}\left(R_{j}\right)=\frac{\partial e_{mag}\left(R_{i},R_{j}\right)}{\partial \varphi_{j}}={\left(R_{j}\frac{m_{j}}{\|m_{j}\|{}_{2}}\;\right)}^{\Lambda }$$

where ${\left(\;\right)}^{\Lambda }$ represents the mapping from SO(3) to so(3).

The Jacobian matrix of $e_{gravity}\left(R_{i},R_{j}\right)$ is:

(32a) $$J_{gravity}\left(R_{i}\right)=\frac{\partial e_{gravity}\left(R_{i},R_{j}\right)}{\partial \varphi_{i}}=-{\left(R_{i}g_{i}\right)}^{\Lambda }$$

(32b) $$J_{gravity}\left(R_{j}\right)=\frac{\partial e_{gravity}\left(R_{i},R_{j}\right)}{\partial \varphi_{j}}={\left(R_{j}g_{j}\right)}^{\Lambda }$$

### 3.4. Loop Closure Constraint Based on Geomagnetic Data

Elimination of cumulative error through loop closure detection has been used in many fields. In the field of visual SLAM, the cumulative error of the visual odometer is significantly eliminated by loop closure detection. In the visual SLAM process, the accurate relative position relationship between the two matched time instants can be obtained after loop detection and this relationship can be directly used as the constraint of the loopback. However, in the loop closure detection based on geomagnetic data, there is only the information that the corresponding positions of the two time instants before and after are similar, rather than a fixed relative relationship. In simple terms, the error function that limits the loopback between moment *i* and moment *j* can be directly defined as:

(33) $$e_{loop}\left(\eta_{i},\eta_{j}\right)=\|\eta_{i}-\eta_{j}{\|}_{2}^{2}$$

where $\eta_{i}={\left[x_{i},y_{i},z_{i}\right]}^{T}$, which represents the coordinate system state at the moment *i*. The corresponding Jacobian matrix is:

(34a) $$J_{loop}\left(\eta_{i}\right)=\left[\begin{array}{c} \left(2\times \left(x_{i}-x_{j}\right)\right)\\ \left(2\times \left(y_{i}-y_{j}\right)\right)\\ \left(2\times \left(z_{i}-z_{j}\right)\right) \end{array}\right]$$

(34b) $$J_{loop}\left(\eta_{j}\right)=-\left[\begin{array}{c} \left(2\times \left(x_{i}-x_{j}\right)\right)\\ \left(2\times \left(y_{i}-y_{j}\right)\right)\\ \left(2\times \left(z_{i}-z_{j}\right)\right) \end{array}\right]$$

As it is hard to avoid mismatches in the loop closure detection, robust Kernel function is required to reduce the impact of mismatches on the overall optimization results. In general, DCS [31] can be selected to improve the overall positioning accuracy in terms of the mismatch problem. Error function with robust Kernel can be expressed as:

(35) $$e_{loopKernel}=\min\left(\frac{2\phi }{\phi +e_{loop}^{2}},1\right)\times e_{loop}$$

where ϕ is the upper limit of deviation. The corresponding Jacobian matrix can be obtained by use of the derivation chained law.

Ideally, the constraint obtained by the direct combination of distance constraint and robust kernel can eliminate the cumulative error. While in practical application, the insufficient discrimination of geomagnetic information leads to a not necessarily reliable match between each independent moment. A more reasonable method is to use a series of loosely matched constraints to confine the position.

**Figure 10:**
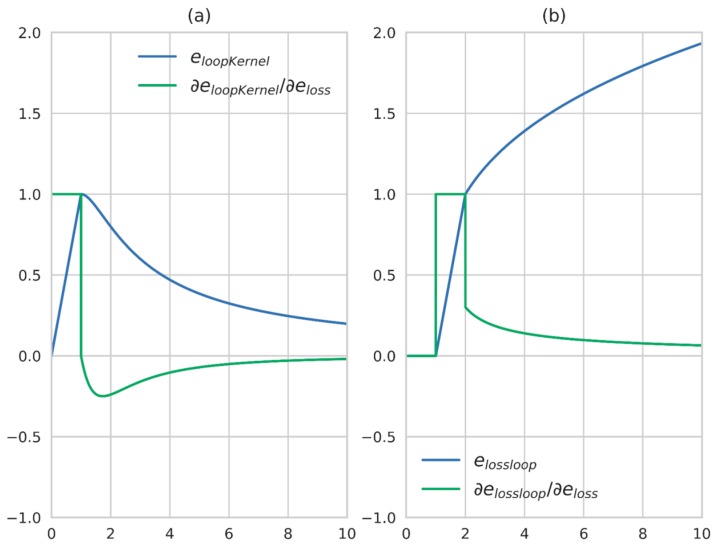
Image of different error functions. (**a**) The function value of different error functions; (**b**) The derivative of different error functions.

The so-called loosely matched constraints, that is, when the distance between two nodes is less than a certain value, the error function value will no longer decrease as the distance decreases. At the same time, considering that the mismatching points may actually be points in the surrounding area, it should not be pushed to a far distance in the optimization process and the right way is to reduce the derivative of the mismatched points and then weaken the importance of the mismatch points in the updating step. Combined the two points above, the error function is defined as:

(36) $$e_{lossloop}=\{\begin{array}{cc} 0 & e_{loop}<\zeta_{low}\;\\ e_{loop}-\zeta_{low} & \zeta_{low}\leq e_{loop}\leq \zeta_{high}\\ {\left(e_{loop}-\zeta_{low}\right)}^{0.3} & \zeta_{high}<e_{loop} \end{array}$$

where $\zeta_{low}$ and $\zeta_{high}$ are model parameters and can be determined according to the actual situation. In the region, less than $\zeta_{low}$, the output of the error function is always 0 and the derivative is also 0, with no effect on the optimization result. In the region between $\zeta_{low}$ and $\zeta_{high}$, the derivative of the cost function is a constant value, with the effect that the distance between two nodes is reduced below $\zeta_{low}$. When the distance is relatively long, the derivative is always positive but gradually reduced and eventually approaching 0. In fact, the situation is the same as using $e_{loopKernel}$ as the error function and only when the distance between two nodes is reduced to a certain extent can the kernel function be activated, otherwise the points with a longer threshold distance will never approach. However, it is hard to determine the right time to activate the kernel only with the algorithm itself. To better illustrate the differences between the two methods, the function value and the derivative of $e_{loopKernel}$ and $e_{lossloop}$, with $e_{loop}$ as the independent variable, are shown in Figure 10. Here $\phi =1.0,\;\zeta_{low}=1.0,\;\zeta_{high}=2.0$.

## 4. Experimental Results and Analysis

### 4.1. Experimental Method

In order to verify the foot-mounted IMU positioning algorithm based on the geomagnetic constraints proposed in this paper, experiments in several indoor scenes were conducted and related data were collected by a wireless IMU based on the MPU9250 chip. The sensor was mounted on the toe to ensure the stability of zero-velocity detection. In the experiment, data of three-axis acceleration, three-axis angular velocity and three-axis magnetometer observation were collected, with a data acquisition frequency of 200 Hz. Regarding the variable motion, the complex path (with a lot of small region motions) and the reverse motion, corresponding data were obtained and further described in Section 4.2.

In this chapter, experimental results are presented and analyzed. The discrimination performance of the magnetic features after FFT and the raw magnetic features are compared in Section 4.2. The deficiency of raw magnetic features is analyzed in Section 4.3 and the performance of RANSAC-based matching algorithm on each data set is also validated. In Section 4.4, the processing effects of error function $e_{loopKernel}$ and error function $e_{lossloop}$ when mismatches exist are compared; besides, the results of the original foot-mounted IMU positioning method and the loop closure constraint method based on magnetometer information are compared.

### 4.2. Comparison between Raw Magnetic Features and Magnetic Features after FFT

In previous studies, matching of geomagnetic feature was always achieved by direct feature processing with DTW algorithm, which is a pretty accurate algorithm for the matching of successive measurements. For the foot-mounted IMU motion, only the magnetometer measurements when foot velocity is 0 can be used for matching and in this condition, although the two walking process pass the same path, the static positions of foot on the ground are not necessarily the same. As a result, errors will be caused by the inconsistency foot positions if direct DTW-based matching is applied. FFT can transform original signals to frequency-domain features and for the processing based on frequency-domain features, the variation of feature distance caused by the slight shift of original signal will be relatively small.

**Figure 11:**
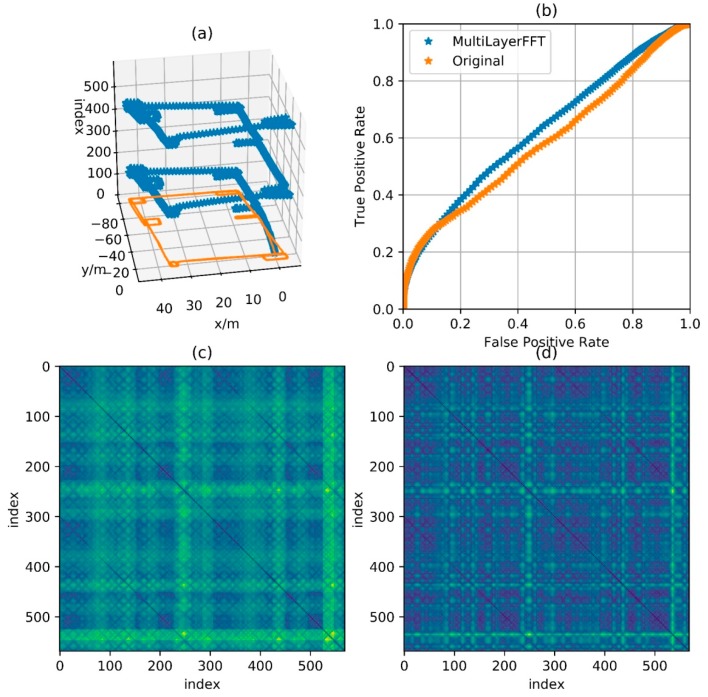
Comparison of performance between BDD and MFD in Path A. (**a**) Reference trajectory; (**b**) ROC of BDD and MFD; (**c**) Distance matrix of BDD; (**d**) Distance matrix of MFD.

**Figure 12:**
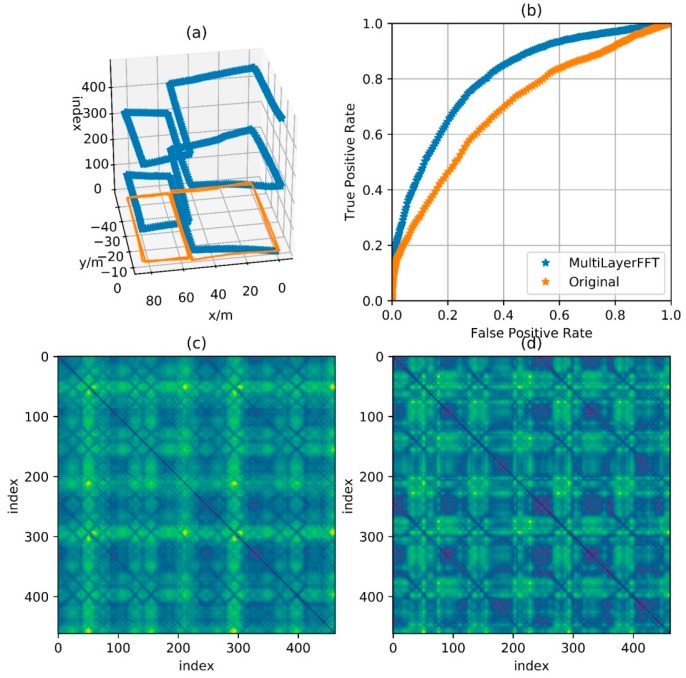
Comparison of performance between BDD and MFD in Path B. (**a**) Reference trajectory; (**b**) ROC of BDD and MFD; (**c**) Distance matrix of BDD; (**d**) Distance matrix of MFD.

**Figure 13:**
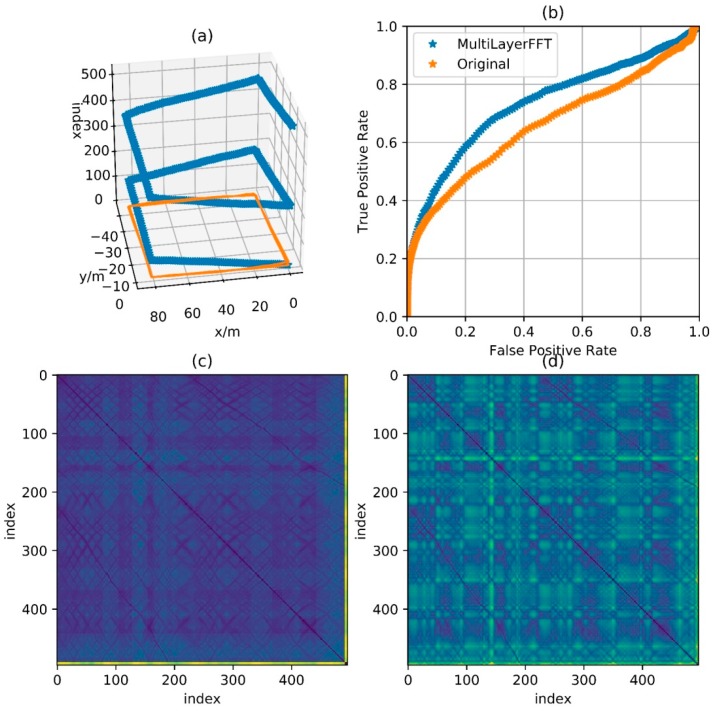
Comparison of performance between BDD and MFD in Path C. (**a**) Reference trajectory; (**b**) ROC of BDD and MFD; (**c**) Distance matrix of BDD; (**d**) Distance matrix of MFD.

**Figure 14:**
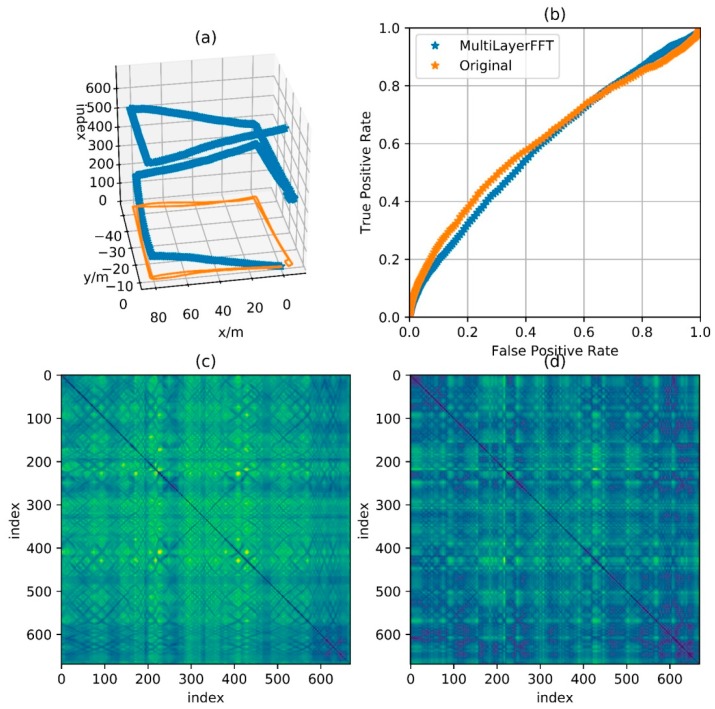
Comparison of performance between BDD and MFD in Path D. (**a**) Reference trajectory; (**b**) ROC of BDD and MFD; (**c**) Distance matrix of BDD; (**d**) Distance matrix of MFD.

Figure 11, Figure 12, Figure 13 and Figure 14 show the reference trajectory, distance matrix and corresponding ROC curve for Path A, B, C and D, respectively. The reference trajectory in sub-graph (a) is constrained by the starting point coincidence, basically showing the real situation of the trajectory. Among them, the orange line is the trajectory projection on the x-o-y plane. For the blue line, the x and y axis values are the trajectory coordinate; the z axis value is the corresponding serial number of the coordinate point in the entire sequence and the serial number increases progressively from the beginning of the walking to the end. In this way, the walking process is intuitively reflected by this blue line. Sub-graph (b) shows the corresponding Receiver Operating Characteristic (ROC) curves for loopback determination with features obtained by BDD and MFD. It reflects the classification quality of the classification algorithm under different parameters and is an important tool to measure the performance of classification algorithm. In this sub-graph, the horizontal axis represents FPR and the vertical axis represents TPR. In fact, the discrimination ability of this algorithm is equivalent to a random guess (50% correct rate in dichotomies) when ROC curves degenerate into a straight line from (0, 0) to (1, 1). The closer the curve is to the upper left corner, the better the algorithm discrimination is. Sub-graph (c) and (d) show the distance matrix obtained by BDD and MFD on each corresponding trajectory, respectively. In fact, it is difficult for the naked eye to distinguish the difference between the distance matrices obtained by the two methods. Note that d=30 m in BBD(i,j,d) and D={5,10,15} in MFD(i,j,D), then the information amount consistency of the original data referred by the two methods is guaranteed and the ability to characterize the original data can be reflected by the performance of the two methods. Here $w_{n}$ and $w_{g}$ are set to 0.5, that is, the total magnetic field strength and the component on the gravitational acceleration direction are considered with the same importance. Comparisons between the two feature distances in the four cases are respectively shown in these four graphs.

Figure 11 shows the feature distance matrix for complex paths. This path is walking around the corridor inside a building for two laps and around six small regions along rectangular track for respective one lap. The path in Figure 12 is walking around the building for two laps along the track with a shape of Arabic numerals “8”, which means that the corridor in the middle is passed by four times. The path in Figure 13 is walking around the floor for one lap with a variable speed. It is clear in the feature matrix that the time instant of the second lap is not aligned with the one of the first lap, that is, points with similar features displayed on the feature distance matrix distribute on a curve instead of a straight line. In fact, the method proposed in this paper is also useful for non-uniform motion, thanks to the mapping from the relationship between feature and time into the relationship between feature and cumulative displacement, as mentioned before. Figure 14 shows a path of reverse-walking, that is, walking around the building for the first lap from the starting point, then around the building in the opposite direction for the second lap and then back to the starting point. During the entire process, walking through a same path in the same direction never happened and all matches are direction-independent match.

According to the ROC curves in Figure 11, Figure 12 and Figure 13, it can be found that the feature distance obtained by MFD is more discriminative than the one obtained by BDD. In Figure 14, both feature distance have similar discrimination. In conclusion, the feature distance obtained by MFD method proposed in this paper presents better performance in most cases.

### 4.3. Sequence Matching Algorithm Based on RANSAC

In fact, both the features collected by MFD and BDD are not enough to be used as matching basis and it can be easily found from the ROC curve. It is hard to find a point on the curve that corresponds to not only a sufficiently low FPR but also a sufficiently high TPR. Therefore, the optimal match is required to be found based on the continuity of sequence data and the RANSAC-based geomagnetic matching algorithm.

**Figure 15:**
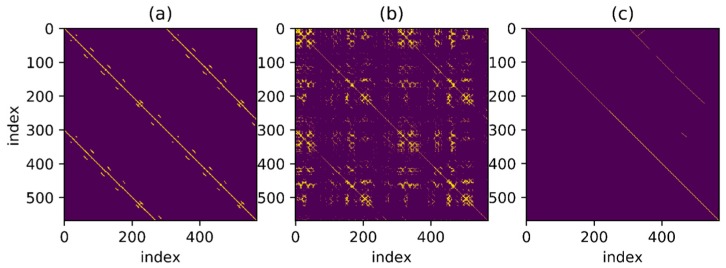
RANSAC-based matching result of Path A. (**a**) Matching relationship generated from the reference trajectory; (**b**) Results when MFD is higher than the threshold; (**c**) Selected matching points based RANSAC.

**Figure 16:**
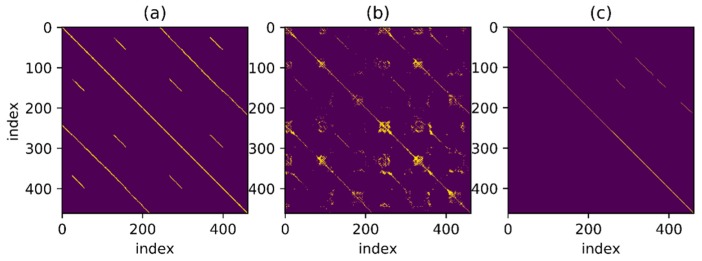
RANSAC-based matching result of Path B. (**a**) Matching relationship generated from the reference trajectory; (**b**) Results when MFD is higher than the threshold; (**c**) Selected matching points based RANSAC.

**Figure 17:**
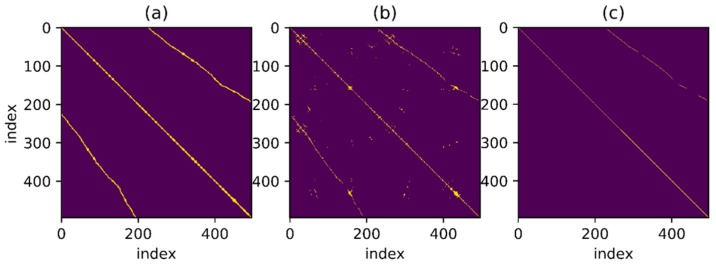
RANSAC-based matching result of Path C. (**a**) Matching relationship generated from the reference trajectory; (**b**) Results when MFD is higher than the threshold; (**c**) Selected matching points based RANSAC.

**Figure 18:**
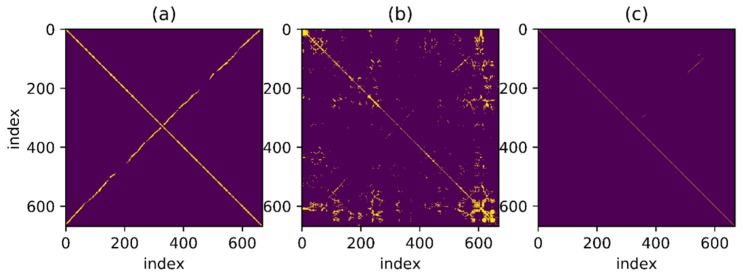
RANSAC-based matching result of Path D. (**a**) Matching relationship generated from the reference trajectory; (**b**) Results when MFD is higher than the threshold; (**c**) Selected matching points based RANSAC.

Figure 15, Figure 16, Figure 17 and Figure 18 show the corresponding RANSAC matching result, reference value and original value for Path A, B, C and D, respectively. Sub-graph (a) shows the matching relationship generated from the reference trajectory, highlighting the points on the trajectory with a distance less than 2.0 m in order to demonstrate the correct match. Sub-graph (b) shows the binary matrix with a selected threshold, highlighting the points where the MFD is less than the threshold. Thresholds are equal in the calculations for all trajectories and the selected points are the input of the RANSAC algorithm. Sub-graph (c) shows the matching points selected by the RANSAC algorithm. The line in the figure is thin and not convenient for observation due to the selected points’ one-to-one matching relationship.

Some thresholds in the RANSAC-based matching algorithm will affect the final result, reasonably set as follows. Set $threshold_{a}$ to 0.1, that is, parameter a in a linear model should fluctuate between 0.9 and 1.1. Set $threshold_{len}$ to 5.0 m, which means that matching sequence smaller than 5 m is not considered as correct matching. Set $threshold_{rate}$ to 0.3, that is, the proportion of outliers after RANSAC algorithm convergence should be less than 0.3. Most of the mismatches can be filtered by the RANSAC-based matching algorithm with parameters setting as above. In the algorithm, a large number of outliers are firstly excluded by the constraint of minimum matching number and the few remaining outliers will not affect the final result. In this way, a large number of incorrect values after the threshold process are excluded. Besides, for the remaining points, the one-to-one matching relationship among a large number of similar points is found by fitting a straight line with the RANSAC algorithm, as explained in the algorithm introduction part.

As can be seen from the figure, the remaining matching points processed by the RANSAC-based matching algorithm are of a high accuracy. Particularly, correct matches are well selected from a connected region in the binary graph by the RANSAC-based sequence matching algorithm, which is hard to be achieved by simply selecting with feature distance. Mismatches are significantly reduced by screening with continuous sequence, resulting in a good one-to-one matching relationship. In sub-graph (c), the main matching parts of Path A, B and C are selected out, while only a small part of the correct matches of Path D are selected out due to the insufficient data discrimination itself. However, mismatches are few in the results of all four paths. Moreover, the above results are long sections of continuous matching and the number of mismatches is relatively small. Considering that the convergence speed of the optimization algorithm heavily relies on the shape of error function, the error function constructed by such a good matching can greatly reduce the computational complexity in subsequent optimization steps and ensure a faster convergence of the algorithm.

### 4.4. Positioning Results Based on Magnetometer Constraint

**Figure 19:**
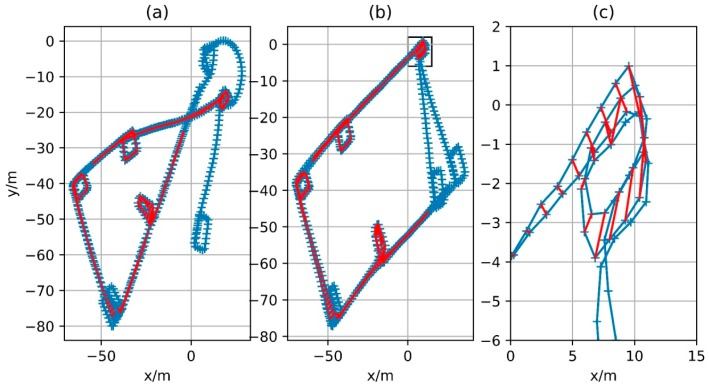
Comparison of results between $e_{loopKernel}$ and $e_{lossloop}$ in Path A. (**a**) Result of $e_{loopKernel}$ adopted error function; (**b**) Result of $e_{lossloop}$ adopted error function; (**c**) Enlarged view of area in (**b**).

**Figure 20:**
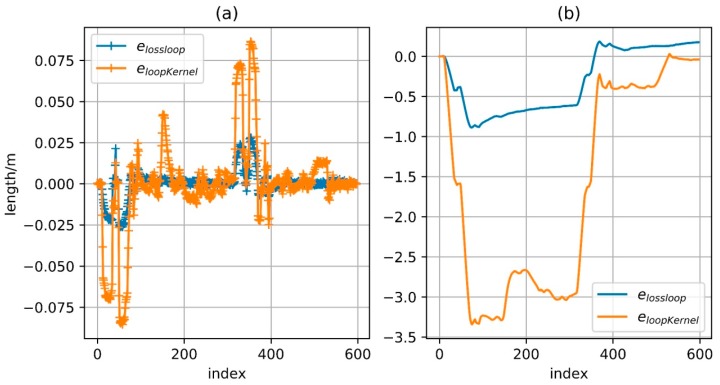
Comparison of step size variation and cumulative variation. (**a**) Difference of the step size between the optimization result and zupt-processed result; (**b**) Cumulative value of step size variation of the optimization result.

The corresponding matching relationship has been obtained based on the geomagnetic features. However, a small number of fault matches or dislocation matches still exist, although the matching relationship during walking is found by the constraints and the RANSAC algorithm. Especially for the dislocation match, it will have a great negative impact on the positioning. The proposed error function $e_{lossloop}$ can reduce the impact of local fault match and dislocation match on the final result. Figure 19 shows the optimization result of the two different error functions. Sub-graph (a) is the result based on the kernel function and the error function $e_{loopKernel}$ with direct distance constraint; sub-graph (b) is the result based on the error function $e_{lossloop}$; Sub-graph (c) is an enlargement of the boxed area in sub-graph (b). The blue line in the figure represents the resulting trajectory and the red line represents the matching result, that is, match is detected at the two time instants connected by the red line. In fact, a simple error function based on kernel function may not conduct optimization because on the initial trajectory, or trajectory obtained by ZUPT, the distance between each matching point is greater than the threshold of the Robust Kernel function. As stated in the previous content, the derivative of $e_{loopKernel}$ outside the threshold region is less than zero, which means that the distance between the two corresponding points is widened. To solve this problem, the error function without robust kernel is directly used for optimization until convergence and then the two error functions are separately used for optimization in order to exclude the initial value’s effect. According to the figure, optimization result processed by the objective function which is constructed by the error function in this paper is much better than the result processed by the error function based on original distance constraint. As can be seen from the sub-graph (c), although there is a certain deviation of the matching result, the distance between the corresponding points is confined to be within a small range by $e_{lossloop}$ and such mismatches will not lead to deformation of the entire trajectory like sub-graph (a). For the single step size, $e_{lossloop}$ also presents a better performance. As in Figure 20, sub-graph (a) shows the difference of the step size between the optimization result under the constraint of error function and the original integral result of foot-mounted IMU algorithm and sub-graph (b) shows the cumulative value of this difference. The error of foot-mounted IMU algorithm based on zero-velocity detection mainly appears in the motion direction and the distance of single-step movement is quite accurate. In fact, there is a high reference value since the cumulative error of velocity has been eliminated by the zero-velocity detection and the error of the step size will not be accumulated over time. There are several large step variations in the optimization result based on the $e_{loopKernel}$ constraint and the cumulative step variation also far exceeds the one in the optimization result based on the $e_{lossloop}$ constraint, which means that the original trajectory scaling is caused by a portion of slightly dislocated matches and the provided final result is not reliable enough. In addition, as $e_{lossloop}$ uses a loose coupling with a longer section of matching, its performance on loop closure constraint is similar to that of $e_{loopKernel}$. So, the relative distance between the corresponding points in each time of loop closure detection is close, which is beneficial for the elimination of the overall cumulative error.

**Figure 21:**
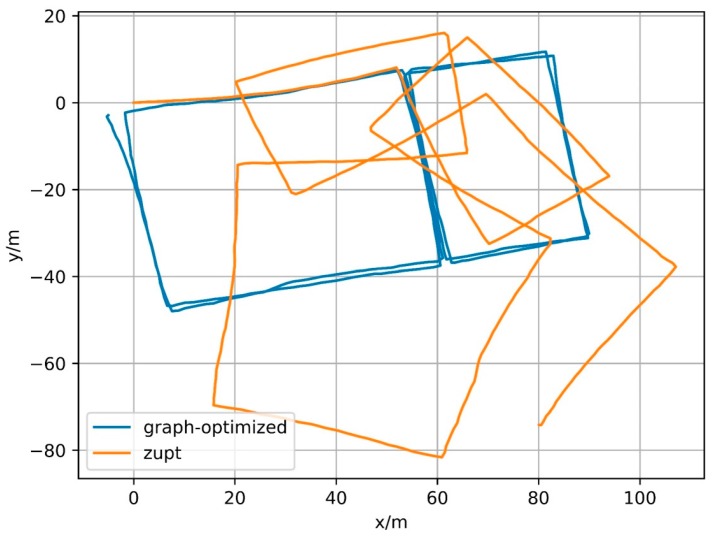
Results of Path B.

**Figure 22:**
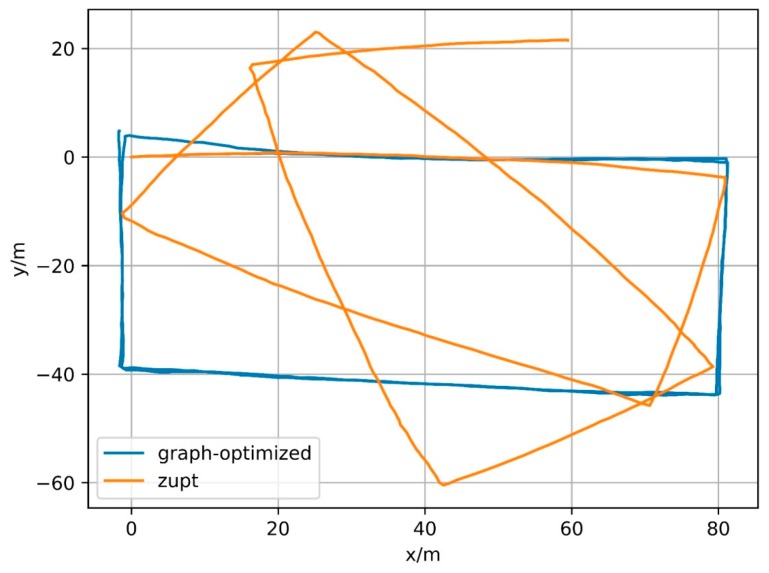
Result of Path C.

**Figure 23:**
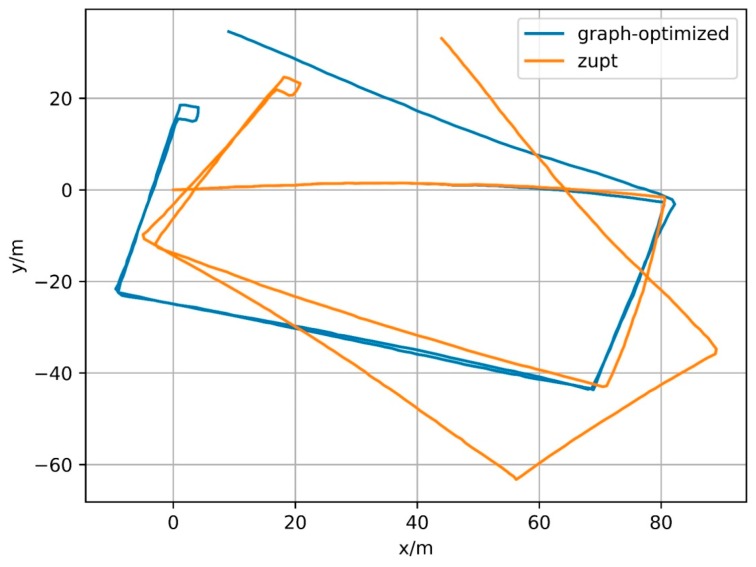
Result of Path D.

In fact, the constraint based on $e_{lossloop}$ also presents good constraint performance for other trajectories. Figure 21, Figure 22 and Figure 23 show the trajectories generated by IMU integral (zupt in the figure) and trajectories generated by process based on magnetometer constraints (graph-optimized in the figure) for Path B, C and D, respectively. The middle part of the trajectories processed by the constraint, that is, both ends of the section contain successfully matching after loop closure detection, can be well coincident. Among them, Path B represents the case of multiple loopbacks, Path C represents the case of variable-velocity walking and Path D represents the case of reverse-walking. The above situations can represent the majority of actual application scenarios. Besides, the positioning method based on foot-mounted IMU benefits from the velocity constraint of zero-velocity detection and its displacement error is relatively small and its positioning error is mainly caused by the cumulative error in the motion direction. After the error in the motion direction is corrected, the positioning result will be of considerable accuracy. In other words, processed by the constraint of geomagnetic features, the positioning algorithm based on foot-mounted IMU have been able to provide a robust positioning result in a large number of scenarios.

In the above results, especially the results of Path A and Path D, the trajectories cannot perfectly coincide on both ends of the loop where no loop constraint is detected. The reason is that features of the short path at both ends of the loop cannot be collected when the information of a long path is used for comparison. In this paper, the largest element in set D is 15 m, so the feature of one point depends on the measured values within 15 m distance before and after, which means that matching cannot be achieved if features of the first 15 m and the last 15 m cannot be collected. Regarding unilateral constraints in practice, even small angular error can lead to large positioning errors after a certain path. Table 1 shows the average positioning errors corresponding to the four trajectories. The global average error represents the average error of the entire trajectory and the constrained-only average error represents the average error of the partial trajectory under loop closure constraint. In practice, the part of trajectory without loop closure constraint is always abandoned to improve the accuracy and reliability of the acquired data. Therefore, the constrained-only average error is more important in practical applications. Among the four trajectories tested in this paper, Path D shows the largest global average error as geomagnetic features are not discriminative enough in reverse-walking and a large section of the path is not constrained by the loopback. Path A presents the largest constrained-only global error due to the mismatches in many parts caused by path complexity. In general, the trajectory accuracy processed by geomagnetic constraint is much higher than the one obtained only by the integral of foot-mounted IMU, the global error of the former is controlled below 3.41 m and the constrained-only average error is controlled below 2.15 m.

## 5. Conclusions

In this paper, a loop closure detection method based on geomagnetic information is proposed for foot-mounted IMU. It can suppress the cumulative error of the positioning algorithm based on IMU integral and can be used as an indoor non-real-time positioning system independent of external base stations. Firstly, the geomagnetic information is expressed as a function of displacement and the performance difference between the feature collected by multi-level FFT proposed in this paper and the feature obtained by direct comparison of original value is compared. Secondly, the correct one-to-one correspondence is found by the RANSAC-based method in the matching with mistake, achieving the loop closure detection. Besides, in order to optimize the positioning result with the information obtained by loop closure detection, a new loop closure constraint error function is designed in this paper. The experimental results show that, combining the feature collection based on multi-level FFT, the loop closure detection based on RANSAC method and the proposed loop closure constraint, the cumulative error of IMU integral on the obtained trajectory is eliminated and a high positioning accuracy is achieved.

## Figures and Tables

**Table 1:** Accuracy of each trajectory.

Trajectory	Global Average Error (m)	Average Error Under Closed Loop Constraint (m)
A	2.75	2.15
B	0.54	0.40
C	1.09	0.96
D	3.41	1.63
